# Targeted inhibition of endothelial calpain delays wound healing by reducing inflammation and angiogenesis

**DOI:** 10.1038/s41419-020-02737-x

**Published:** 2020-07-14

**Authors:** Chenlong Yi, Weihua Wu, Dong Zheng, Guangying Peng, Haoyue Huang, Zhenya Shen, Xiaomei Teng

**Affiliations:** 1 Department of Cardiovascular Surgery of the First Affiliated Hospital & Institute for Cardiovascular Science, Soochow University, 215006 Suzhou, China; 2 Center of Clinical Laboratory, The First Affiliated Hospital of Soochow University, 215006 Suzhou, China

**Keywords:** Cellular imaging, Inflammation

## Abstract

Wound healing is a multistep phenomenon that relies on complex interactions between various cell types. Calpains are a well-known family of calcium-dependent cysteine proteases that regulate several processes, including cellular adhesion, proliferation, and migration, as well as inflammation and angiogenesis. CAPNS1, the common regulatory subunit of Calpain-1 and 2, is indispensable for catalytic subunit stabilization and activity. Calpain inhibition has been shown to reduce organ damage in various disease models. Here, we report that endothelial calpain-1/2 is crucially involved in skin wound healing. Using a mouse genetic model where *Capns1* is deleted only in endothelial cells, we showed that calpain-1/2 disruption is associated with reduced injury-activated inflammation, reduced CD31^+^ blood vessel density, and delayed wound healing. Moreover, in cultured HUVECs, inhibition of calpain reduced TNF-α-induced proliferation, migration, and tube formation. Deletion of *Capns1* was associated with elevated levels of IκB and downregulation of β-catenin expression in endothelial cells. These observations delineate a novel mechanistic role for calpain in the crosstalk between inflammation and angiogenesis during skin repair.

## Introduction

The skin is the largest organ of the human body and acts as a protective barrier against external stimuli and pathogen invasion. In most cases, acute injuries initiate an immediate emergency response to establish a repair and regeneration environment^1^. Skin wound healing undergoes multiple overlapping phases, including a peak angiogenic response after injury, followed by decreased vessel numbers upon wound closure and simultaneously fibrosis and scarring^2^. These repair mechanisms involve in elaborate and intricate processes and remain incompletely understood^3^.

Calcium serves as a modulator in the normal homeostasis of mammalian skin. In wound repair, calcium is predominantly involved as Factor IV in the hemostatic phase, but it is indispensable in cell migration and regeneration patterns in later stages of healing^4^. Calpains belong to a family of calcium-dependent cysteine proteases^5^, which have the unique property of modifying the activity/function of their substrates by protein cleavage. Among 16 family members, calpain-1 and calpain-2 are two most extensively studied isoforms, and are expressed ubiquitously in mammals and many other organisms. Of note, calpain-1 and calpain-2 are the only known calpain isoforms expressed in endothelial cells (ECs)^6^. Calpain-1/2 isoforms are heterodimers consisting of CAPN1 and CAPN2 catalytic subunits, respectively, and the common small regulatory subunit CAPNS1, also known as CAPN4. These ubiquitous classical calpain isoforms are regulated by the endogenous calpain-specific inhibitor, calpastatin (CAST). Inhibiting calpain activity has been shown to reduces tissue damage in experimental models of inflammatory diseases^7^, cancer progression^8–10^, neurodegeneration^11^, and cardiovascular diseases^12^. Calpain activity also appears to be essential in skin wound healing^13^. Loss-of-function mutations in calpastatin cause PLACK syndrome, which is characterized by multiple skin pathologies^14^. CAST overexpression strikingly delays wound healing with reduced proliferation and re-epithelialization^13^. Whereas selective inhibition of calpain-1 in diabetes reverses dermal defects and improves the quality of diabetic skin after wound healing^15^.

Skin wound healing is a complex process. The wound site is usually subject to hypoxic conditions during healing, which results in sprouting angiogenesis. This event is crucial for proliferating tissues and creating an access route for inflammatory cells. In vascular endothelial cells, calpains have been reported to be associated with sprouting angiogenesis^16^. We reported that inhibition of calpain reduced oxidative stress and attenuated endothelial dysfunction in diabetes^17^. Endothelial cell-specific deletion of *Capns1* reduced diabetic cardiomyopathy by improving angiogenesis^18^. These findings indicate a critical role of endothelial calpain in microvascular endothelial dysfunction. However, these causes of endothelial dysfunction that delays skin wound healing remain incompletely understood. Calpain activation has been implicated in endothelial dysfunction and inflammation. This raises an intriguing possibility that calpain-mediated endothelial injury may contribute to skin wound healing.

In this study, we investigated the role of endothelial calpain in skin wound healing using mice with endothelial cell-specific deletion of *Capns1*.

## Results

### Deficiency of endothelial cell *Capns1* delays wound healing of dorsal skin

**Fig. 1:**
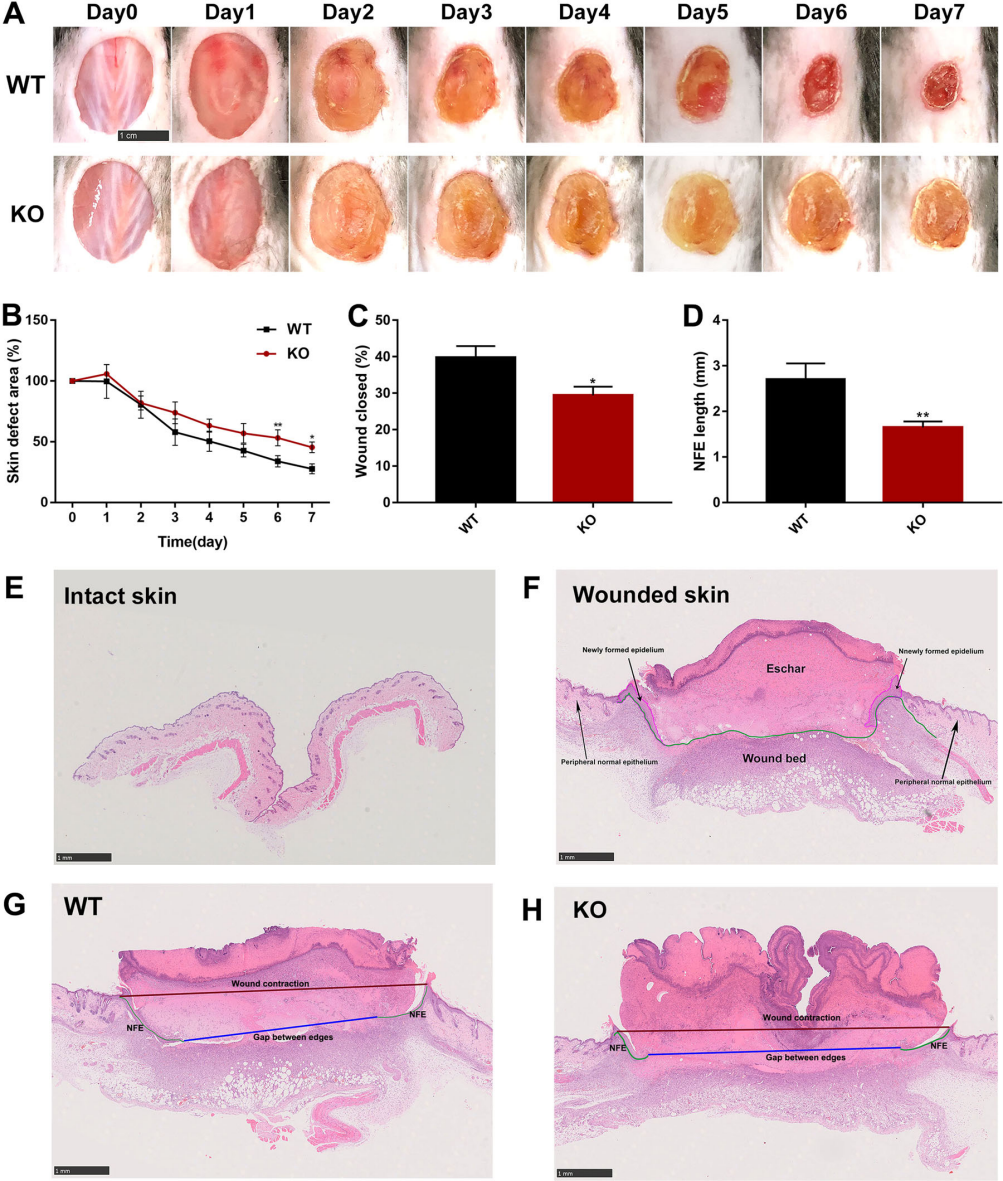
Deletion of endothelial *Capns1* delayed wound healing in mice. **a** Representative wounds from WT or KO mice were shown (scale bar, 1 cm). **b** Statistical analysis of wound areas were shown as the percentage of each timepoint in comparison to the initial (day 0) wound size. **c**–**h** Morphological changes in the wounds of the mice (scale bar, 1 mm). H&E-stained sections described the intact skin (**e**) and a full-thickness excisional wound of WT mice at day 7 postsurgery (**f**). Newly formed epithelium (NFE, pink areas) and wound bed (areas under green line) were present in **f**. Based on this, the percentage of wound closure [length of NFE(green line)/length of NFE+ length of gap between edges of wound epithelium (blue line)] and re-epithelialization (length of NFE) in WT mice (**g**) and KO mice (**h**) at D7 were carried out for morphometric analyses (**c**, **d**). (Data are the mean ± SD, *n* = 5 in each group, *P < 0.05, **P < 0.01, vs WT mice).

To evaluate the effects of endothelial cell-specific calpain-1/2 disruption following skin injury, full-thickness 15 mm diameter excisional wounds were made on TEK-CRE^+/−^ *Capns1*^PZ/PZ^ (KO) mice and their wild-type (WT) littermates, and the wounds were monitored for 7 days. Although the dynamics of wound contraction can be variable, as previously described^19,20^, we found a significant delay in wound healing of KO mice compared with that of WT mice (Fig. 1a). A trend toward reduced wound closure in KO mice became apparent at 3 days and was significantly different by 6 days (Fig. 1b). The mean wound areas of WT mice were reduced to 28.48% of the original wound size at 7 days, while those of KO mice remained at 45.44% of the original injured areas (Fig. 1b). Morphological abnormalities were profound with impaired re-epithelialization and granulated tissue formation in KO mice compared to those of WT mice. Morphometric analyses of wound sections (Fig. 1c–h) displayed a delay in wound closure in KO mice by quantification of the distance of newly formed epidermis covering the wound, an average of 39.74% of the wound was covered by epidermis in control mice, while only 29.40% of the wound was covered in *Capns1*-KO mice at the same timepoint (Fig. 1c, g, h). Likewise, the length of the new formed epithelium was significantly decreased (1.65 ± 0.13 mm in KO vs. 2.71 ± 0.34 mm in WT mice) (Fig. 1d, g, h). These results suggest that wound healing is substantially delayed as a result of calpain-1/2 disruption in endothelial cells.

### Deletion of calpain in endothelial cells hinders the wound inflammatory response and angiogenesis

**Fig. 2:**
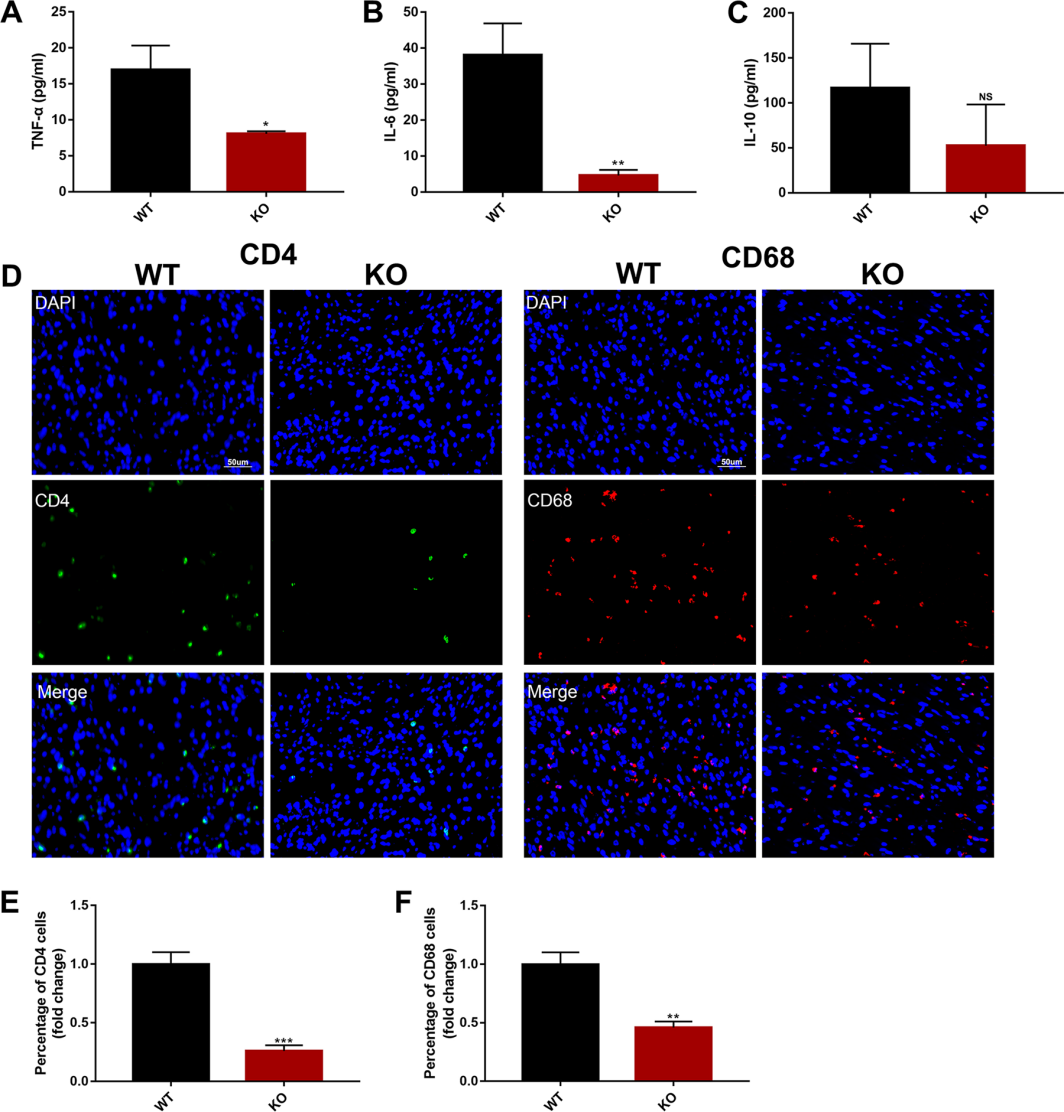
Deletion of endothelial *Capns1* prevented the inflammatory responses in skin wounds. **a**–**c** The levels of serum TNF-α, IL-6, and IL-10 of mice at day 7 after injury were detected by ELISA. **d**, **e** Immunofluorescence images showing CD4^+^ T lymphocyte (gree) and CD68^+^ macrophage (red) infiltration in the wound beds. (scale bar, 50 μm). **e**, **f** Quantitative data of cell infiltration (data are the mean ± SD *n* = 5 in each group *P < 0.05, **P < 0.01, ***P < 0.001, vs WT mice).

The inflammatory phase is the inevitable stage of skin wound healing. To explore the potential role of calpain-1/2 in this inflammatory phase, we compared inflammatory cytokines from peripheral blood and infiltration of inflammatory cells in skin sections from 7-day-old excisional wounds of WT and KO mice. Serum TNF-α and IL-6 levels were significantly higher in the WT mice than KO mice (TNF-α: 8.06 ± 0.20 pg/mL in KO vs. 16.98 ± 1.93 pg/mL in WT mice, IL-6: 1.175 ± 0.28 pg/mL in KO vs. 12.16 ± 1.53 pg/mL in WT mice) (Fig. 2a, b). There was no significant difference in IL-10 serum levels between KO and WT mice (Fig. 2c). We also observed reduced numbers of CD4^+^ T lymphocytes and CD68^+^ macrophages in the wound beds of *Capns1-*KO mice compared to those of WT mice (Fig. 2d–f), but no changes in the number of CD8^+^ T lymphocytes or CD45^+^ neutrophils (data not shown). These data indicate that deletion of calpain in endothelial cells reduces the inflammatory response in wounds.

**Fig. 3:**
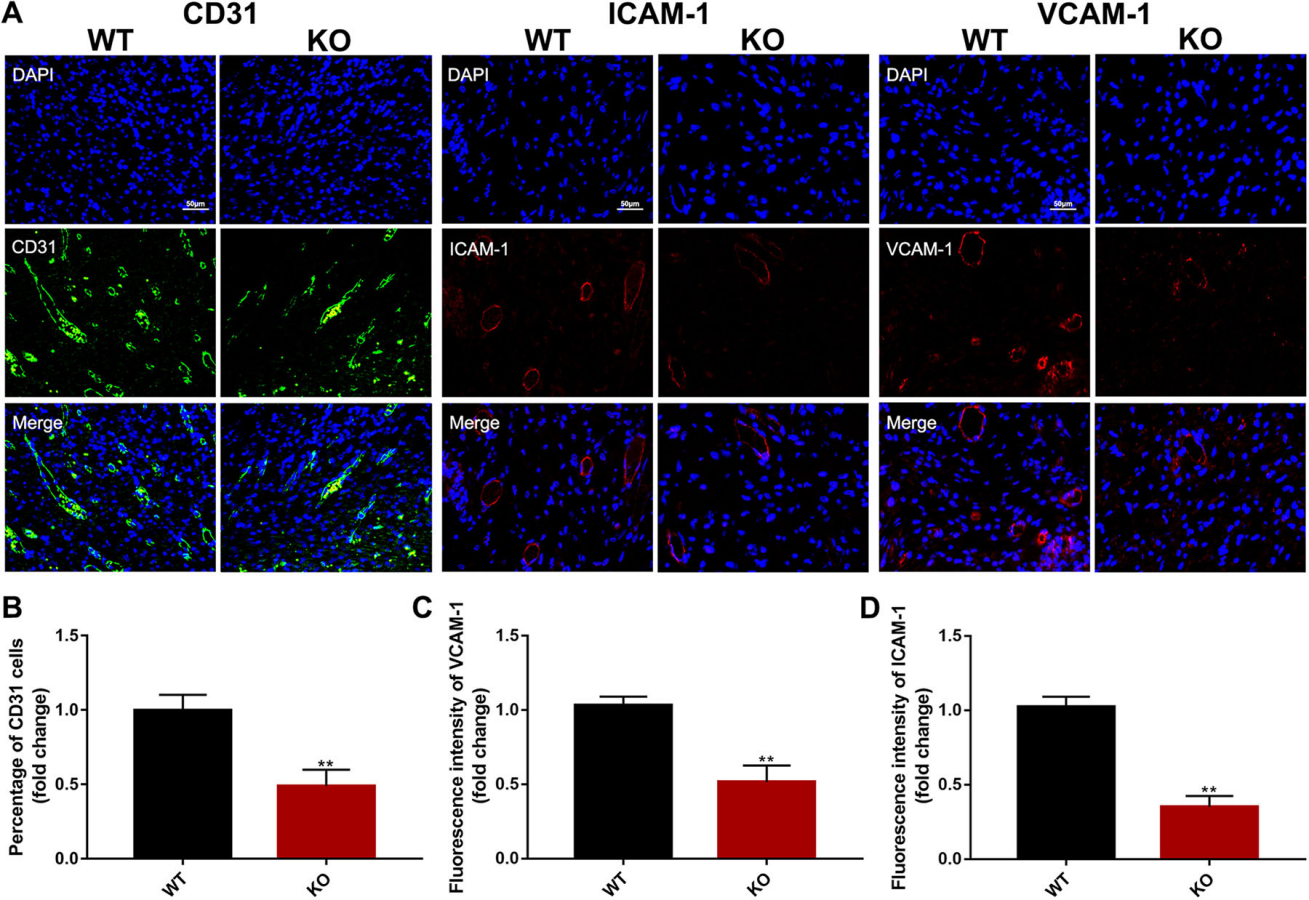
Calpain promoted angiogenesis and endothelial cell activation at skin wound sites. **a** Seven days after injury, the wound sites were removed. Endothelial activation and vessel density were assessed by immunolabelling CD31 (marker of endothelial cells) (green) and ICAM-1 and VCAM-1 (markers of activated endothelial adhesion molecules) (red). **b–d** Quantification of CD31^+^ cells, and expression of ICAM-1 and VCAM-1 in skin wounds. (*n* = 5 **P < 0.01, vs WT mice).

CD31 staining of wound sections revealed a significant reduction in blood vessel density in KO relative to WT mice (Fig. 3a, b). Inflammatory cells are known to produce growth factors, cytokines, and chemokines that can act on vascular endothelial cells and can upregulate expression of adhesion molecules including ICAM-1 and VCAM-1. Consistent with the observed reduction in inflammatory cytokine expression, the expression of these endothelial adhesion molecules was reduced in wounds of KO relative to WT mice (Fig. 3a, c, d). Given the role of adhesion molecules in leukocyte recruitment, it is possible that the observed reduction in recruitment of CD4^+^ and CD68^+^ cells is associated with reduced endothelial cell adhesion molecule expression. Together, these findings indicate that endothelial cell expression of calpain-1/2 promotes skin wound healing, possibility through effects on endothelial cell proliferation and inflammatory functions.

### Inhibition of calpain prevents inflammatory response to TNF-α in HUVECs

**Fig. 4:**
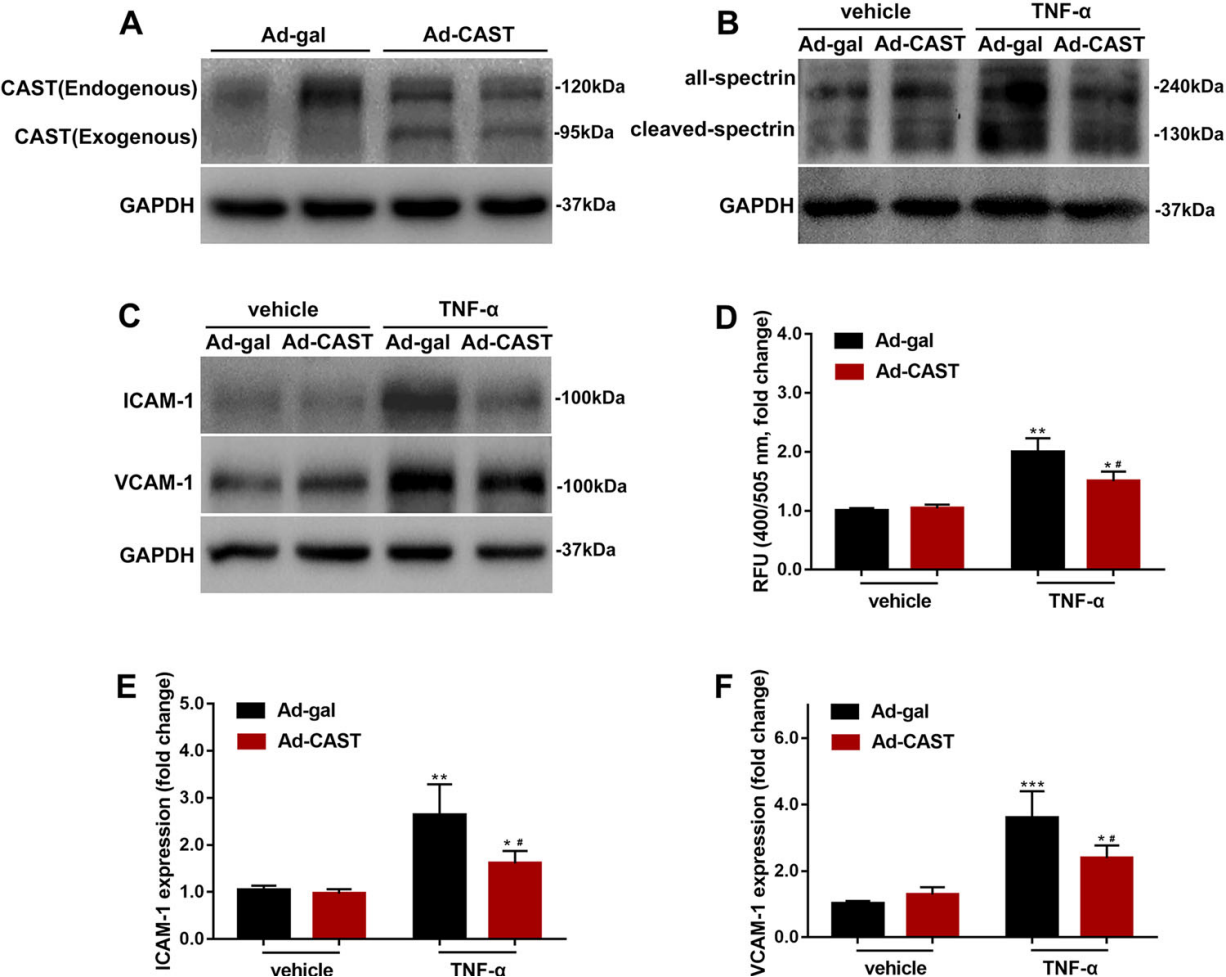
Transfection of Ad-CAST inhibited the endothelial cell response to TNF-α stimulation. Cultured HUVECs were infected with Ad-CAST or Ad-gal as a control. After treatment with TNF-α (10 ng/mL) for 6 h, the protein levels of calpastatin, αII Spectrin, ICAM-1, VCAM-1, and GAPDH were determined by western blotting. **a**–**c** Representative western blots were shown. **d** Calpain activity was elevated as measured a fluorescence substrate, Ac-LLY-AFC. **e** ICAM-1 protein levels relative to GAPDH. **f** VCAM-1 protein levels relative to GAPDH. (data are the mean ± SD, *n* = 5 independent cell batches. **P < 0.01, ***P < 0.001, vs Ad-gal + vehicle; ^#^*P* < 0.05, vs Ad-gal + TNF-α).

Angiogenesis and inflammation are closely integrated processes in a number of physiological and pathological conditions^21,22^. Inflammatory cells can directly release angiogenic factors, such as VEGF, ANG, bFGF, TGF-β, and TNF-α, thereby exerting mitogenic and migratory effects on the endothelium^23,24^. TNF-α, as the main stimulus to mimic the inflammation environment in host tissue, may increase intracellular calcium concentration in macrophages and cardiomyocytes^25,26^. To explore the role of calpain on endothelial cell activation induced by proinflammatory factors, we infected human umbilical vascular endothelial cells (HUVECs) with adenovirus expressing the calpain-1/2 inhibitor CAST or b-galactosidase (Ad-CAST or Ad-gal, respectively), and then incubated the cells with TNF-α (10 ng/mL) to mimic inflammation in vitro or vehicle for 6 h (Fig. 4a, b). Calpain activity was assessed by the cleavage of endogenous spectrin (a well-characterized calpain substrate), and quantified in cell lysates using the fluorescent substrate Ac-LLY-AFC. TNF-α treatment enhanced calpain activity (Fig. 4b–d), and stimulated the expression of the adhesion molecules ICAM-1 and VCAM-1 in HUVECs (Fig. 4c, e, f). Overexpression of calpastatin by Ad-CAST transfection inhibited calpain activity and activation of endothelial cells.

### Inhibition of calpain prevents proliferation and angiogenesis in HUVECs under inflammatory conditions

**Fig. 5:**
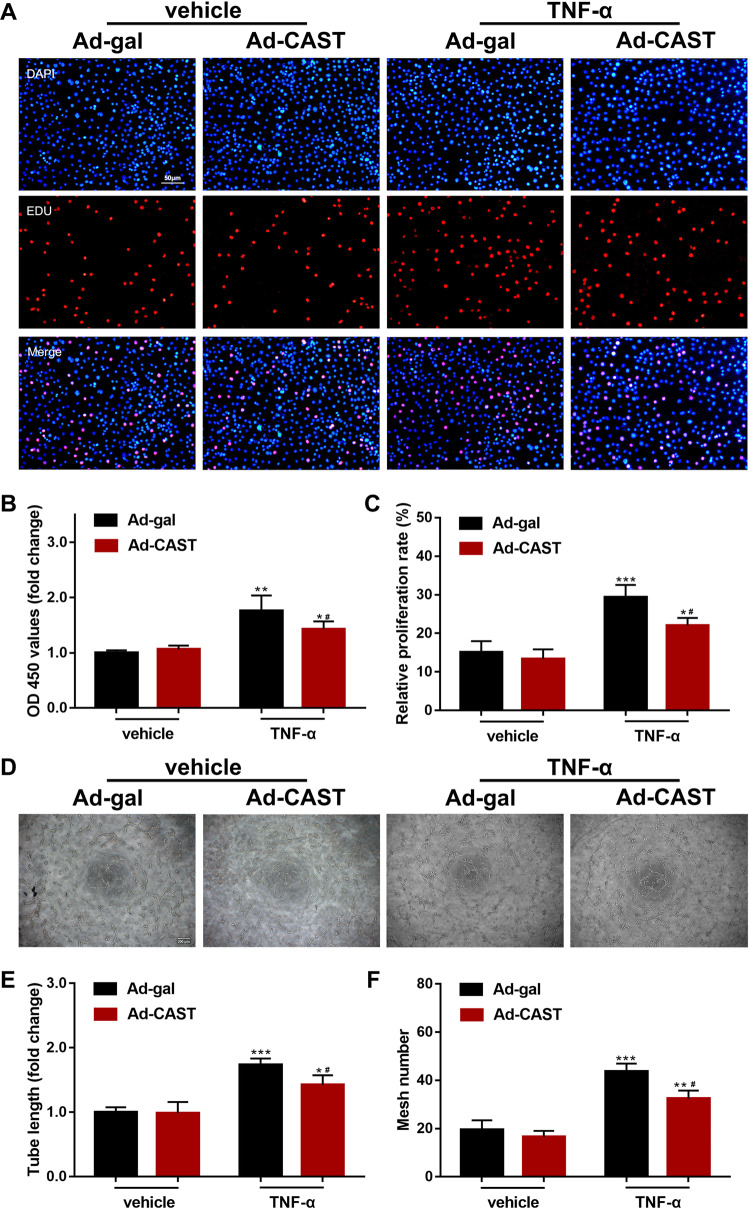
Overexpression of CAST impaired cell viability, proliferation, and tube formation. Cultured HUVECs were infected with Ad-CAST or Ad-gal as a control in the presence of TNF-α (10 ng/mL) or vehicle. **a** A representative micrograph of endothelial proliferation via EdU assay (nuclear staining with DAPI, blue; proliferating cells stained with EdU, red) (scale bar, 50 μm). **b** Cell viability was detected by CCK-8 assay. **c** Statistical results for the EdU assay. **d** A representative micrograph for tube formation (scale bar, 200 μm). **e**, **f** Quantitative data of endothelial cell tube formation, as determined by tube length and mesh number (data are the mean ± SD, *n* = 5 independent cell batches. ***P < 0.001, vs Ad-gal + vehicle; ^#^*P* < 0.05, vs Ad-gal + TNF-α).

**Fig. 6:**
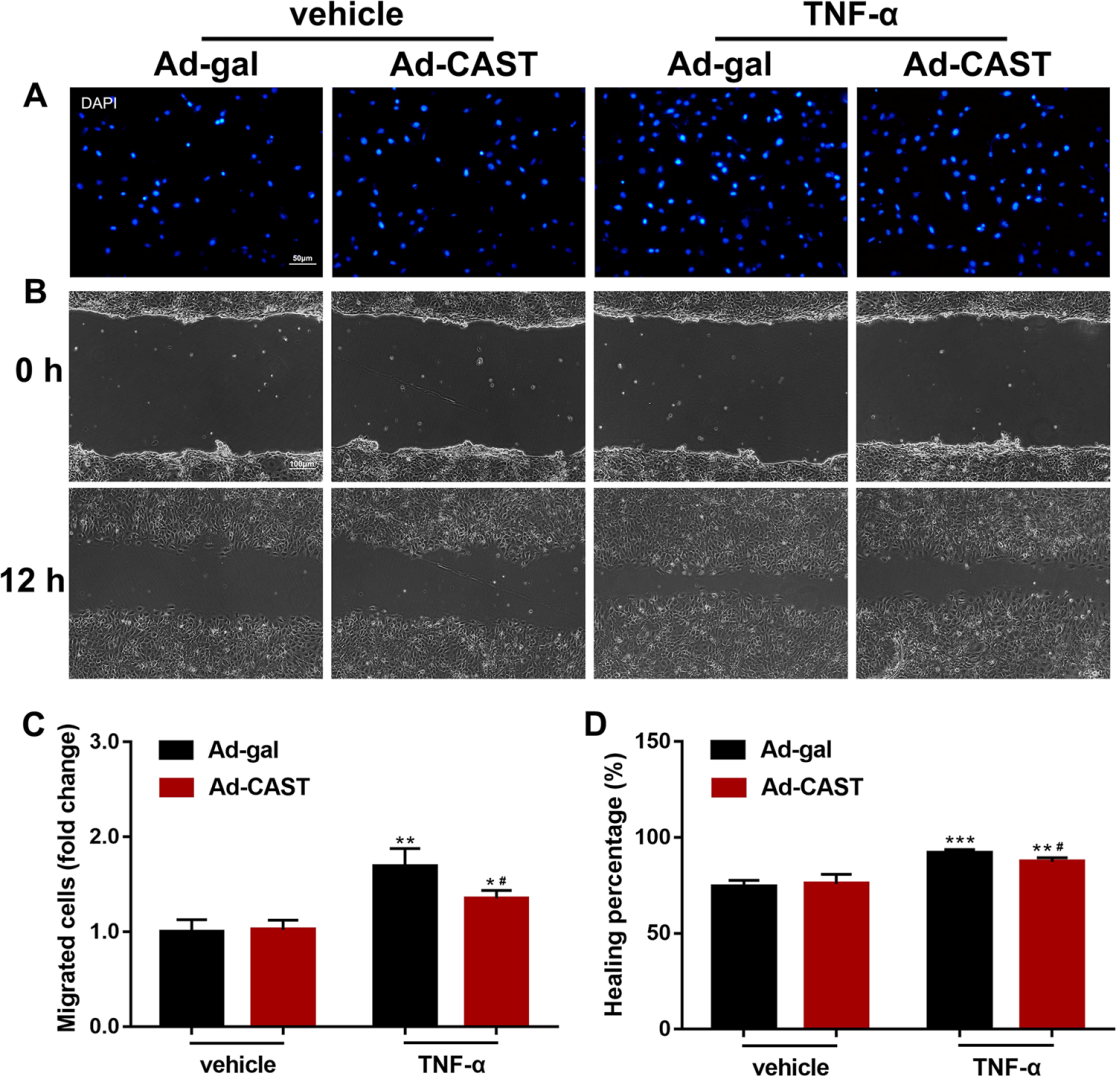
Overexpression of CAST attenuated TNF-α-induced cell migration and healing. **a** A representative micrograph for migration of endothelial cells (scale bar, 50 μm). **b** A representative micrograph for wound healing (scale bar, 100 μm). Quantitative data for migrated cells (**c**) and the percentage of healing (**d**) (data are the mean ± SD, *n* = 5 independent cell batches. ***P < 0.001, vs Ad-gal + vehicle; ^#^*P* < 0.05, vs Ad-gal + TNF-α).

Immune cells circulating though the microvasculature and extravasating into inflamed tissue contribute to angiogenesis by inducing migration, survival, and proliferation of endothelial cells during the formation of new vasculature^27^. To investigate whether endothelial cell response to inflammatory stimulation was regulated by calpain, we assessed cell viability and proliferation (using CCK-8 and EdU assays, respectively), as well as migration and angiogenesis (using transwell or wound healing and tube formation assays, respectively) in HUVECs infected with Ad-CAST or Ad-gal after TNF-α (10 ng/mL) or vehicle treatment for 6 h. TNF-α promoted the proliferation, migration, and tube formation in HUVECs, but these effects were attenuated by infection with Ad-CAST (Figs. 5, 6). Consistently, incubation with Calpain inhibitor I (ALLN) achieved similar negative effects in HUVECs (Supplementary Figs. 2, 3). These results demonstrate that calpain promotes angiogenesis behavior of endothelial cells in response to TNF-α-induced inflammation signaling.

### CAST overexpression enhanced IκB expression and suppressed β-catenin protein in endothelial cells

**Fig. 7:**
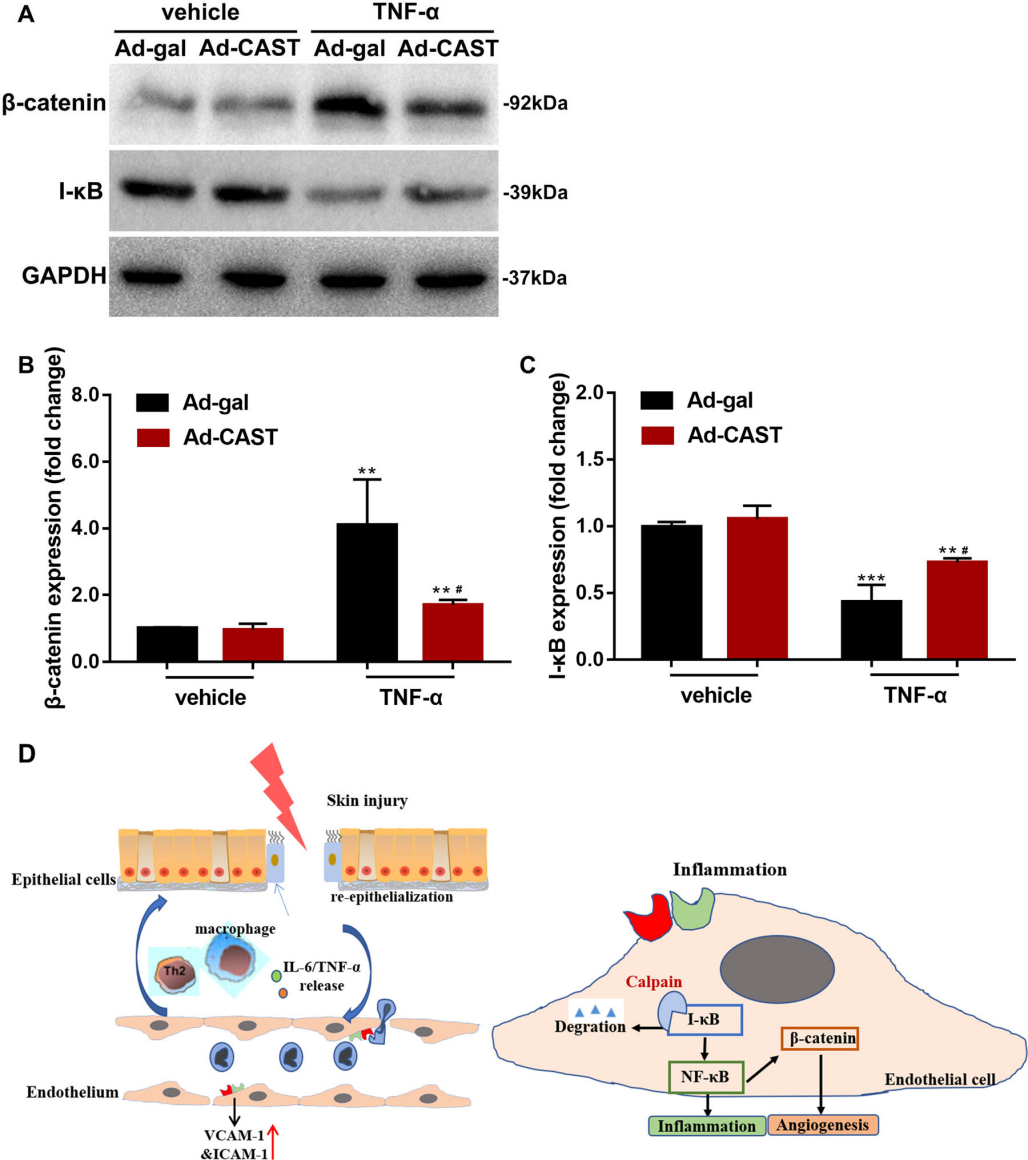
CAST overexpression modified HUVEC expression of β-catenin and IκB in response to TNF-α challenge. **a** Western blot analysis using anti-β-catenin and anti-IκB antibodies. **b** β-catenin protein levels relative to GAPDH. **c** IκB protein levels relative to GAPDH (data are the mean ± SD from at least four independent experiments. **P < 0.01, ***P < 0.001, vs Ad-gal + vehicle; ^#^*P* < 0.05, vs Ad-gal + TNF-α). **d** Schematic illustration of wound healing by endothelial calpain systems. Skin injury activates adhesion molecules in ECs, such as VCAM-1 and ICAM-1, and triggers the subsequent release of chemokines and inflammatory cell infiltration. Calpain activity stimulates NF-κB pathway via IκB cleavage under inflammatory conditions, and indirectly mediates β-catenin, leading to the process of healing. Transgenic knockout of *capns1* in ECs prevents NF-ΚB signaling, delays skin wound repair.

Calpain has been reported to cleave β-catenin and activate nuclear factor kappa-B (NF–κB) through the proteolysis of IκB in the context of angiogenesis and inflammation^28,29^. We therefore examined the effect of CAST overexpression on β-catenin and NF−κB in HUVECs challenged with TNF-α. We showed that TNF-α induced β-catenin and suppressed IκB expression in HUVECs, and these effects were both attenuated by infection with Ad-CAST (Fig. 7).

## Discussion

In the present study, we demonstrated a protective role of endothelial calpain-1/2 during skin wound healing. In endothelial-specific *Capns1*-KO mice, wound healing was strikingly delayed (Fig. 1). During re-epithelialization, the density of the inflammatory infiltrates and blood vessels were reduced in KO mice. This was in accordance with in vitro data showing that calpain inhibition led to a reduction in proliferation, migration, and tube formation in HUVECs in response to proinflammation induction with TNF-α. The deleterious effects of calpain deletion were associated with an upregulation of IκB and the downregulation of β-catenin during wound repair.

CAPNS1 acts as an obligate regulatory subunit for calpain-1 and calpain-2 by forming a heterodimer with their respective unique 80 kDa large catalytic subunits^30–32^. Studies have shown that *Capns1* expression is associated with oncogenesis in solid tumors^32,33^. *Capns1* knockdown or knockout in breast carcinoma cells results in markedly decreased migration and invasion in vitro and metastatic activity in vivo in preclinical mouse models^9,10,34^. Other studies showed that calpain activity in endothelial cells was induced by VEGF, IL-6, or shear stress, and was involved in angiogenesis in skin wound healing^18,35^. Although the exact mechanisms by which calpain in endothelial cells improves repair of wounded skins are currently unknown, our findings suggest that roles in endothelial cell inflammatory signaling, and improved angiogenic responses may be important in this scenario.

Vascular ECs are located on the surface of the vascular endothelium, forming a barrier between circulating blood and local tissues. When ECs are damaged or exposed to inflammatory signals, such as TNFs and endotoxins, a series of metabolic and structural changes occur. This process is called endothelial cell activation. Activated endothelial cells increase the secretion of many cytokines, such as IL-1, IL-6, IL-10, and platelet activating factor, and the expression of cell surface adhesion molecules, including ICAM-1 and VCAM-1. These molecules induce circulating inflammatory cells to roll along, adhere to and penetrate the endothelial surface, and further migrate into local tissues. Recruited leukocytes can promote angiogenesis by release of endothelial growth factors^36,37^. Thus, inflammation is often associated with increased angiogenesis.

Prior experiments and our data showed that sustained inflammation activates vascular endothelial cells, leading to sustained high expression of adhesion molecules and chemokines^38^. Inflammation including infiltration of CD4^+^ T lymphocytes and CD68^+^ macrophages into the wound sites and high levels of serum TNF-α and IL-6 were shown in the skin wound model (Fig. 2), accompanied by remarkable increases of adhesion molecules and CD31^+^ vessel density (Fig. 3). Neutrophils are the first immune cells to the wound bed and remain for about 24 h before undergoing apoptosis, helping to control infection and remove debris after tissue injury^39,40^. Cytokines released by neutrophils during apoptosis are chemotactic for monocytes and lymphocytes. These monocytes differentiate into macrophages, which can remain for several weeks at the wound site. Macrophages secrete chemokines to attract T cells to the wound site and encourages CD4^+^ T helper polarization^41^. Apoptotic neutrophils are phagocytosed by the newly arrived macrophages. This could explain why reduced T lymphocytes and macrophages were observed in 7-day-old excisional wounds of KO mice, but neutrophil numbers were unaffected in this study.

Using an in vitro model, we showed that the inflammatory factor TNF-α enhanced ICAM-1 and VCAM-1 expression, and promoted HUVECs proliferation, migration, and tube formation via activated calpain-1/2 activity (Figs. 4, 5). Inhibition of calpain blocked these proangiogenic phenotypes, in accordance with calpain participating in the activity and motility of endothelial cells^42^. NF-κB is an important nuclear transcription factor in the acute and chronic inflammatory response. Inhibition of NF-κB prevents the activation of endothelial cells though the overexpression of IκB, an endogenous inhibitor of NF-κB^43,44^. Calpains have also been reported to be modulators of the NF-κB signaling pathway via the degradation of IκB^45^, which was verified in our experiments. Thus, endothelial calpain-1/2 might contribute to inflammation through effects on the NF-κB signaling pathway.

Endothelial calpain systems are also responsible for angiogenic regulation. Our previous study showed that deletion of endothelial cell *Capns1* in mice reduced diabetic cardiomyopathy by improving angiogenesis induced by increased β-catenin protein levels^18^, which is consistent with the β-catenin-mediated improvement in angiogenesis in several studies^29,46^. In contrast, the present study demonstrated that endothelial calpain activity promotes angiogenesis in skin wound repair and HUVECs under TNF-α stimulation. Intriguingly, calpain-1/2 inhibition reduced the TNF-α -induced protein level of β-catenin. This mechanism may be related to crosstalk between Wnt/β-catenin and the NF-κB signaling pathways during inflammation. Several studies have reported a positive regulation of Wnt/β-catenin signaling by the NF-κB pathway. IKKα and IKKβ, the critical activators of the NF-κB pathway, are also involved in the regulation of β-catenin-dependent transcriptional activity^47–49^. Some studies support that calpain activity is required for wound healing, transgenic mice with overexpressed CAST, which displayed a striking delay in skin wound healing through impaired angiogenesis^13,50^. These differences imply that calpain-1/2 modulates angiogenesis via multiple mechanisms, depending on the environment and tissues. However, the underlying molecular mechanisms of this finding remain to be elucidated.

In conclusion, our study sheds new light on the roles of endothelial calpain in skin wound healing through inflammation-induced angiogenesis. Calpain-1/2 inhibition impedes inflammatory cell recruitment and angiogenesis, resulting in delayed wound healing and cell injury. Therefore, the effects of modifying calpain activity on inflammation, wound healing and skin regeneration should be carefully considered in the context of future therapeutic applications.

## Materials and methods

### Animals

All experimental procedures were approved by the Animal Use Subcommittee of Soochow University (Suzhou, China). C57BL/6 mice and TEK-CRE^+/−^ mice were purchased from Jackson Laboratory (Sacramento, CA, USA). Mice with endothelial cell-specific *Capns1* knockout (KO) were generated by breeding *Capns1* floxed (Capns1^PZ/PZ^) and TEK-CRE^+/−^ mice as previously described^18^. The breeding program was implemented at the animal care facilities of Soochow University’s.

Genotyping was detetmined by PCR, using gene-specific primers (Supplementary Table1 and Supplementary Fig. 1).

### Dorsal excisional wound model

The mice were anesthetized by inhalation of isoflurane, and full-thickness excisional skin wounds were made in the dorsal skin using a sterile biopsy punch with a diameter of 15 mm (Kai Industries, Tokyo, Japan). Wounds were left uncovered until they were harvested. After surgery, the dorsal wounds were photographed every day using a Sony CybershotH 10.1 megapixel DSC-W180 digital camera. Changes in wound areas were expressed as the proportion of the initial wound areas. Subsequently, the wounds and their surrounding areas, including the scab and epithelial margins, were harvested for further analysis. All quantifications were performed on tissue samples obtained from males only. The experimenters were not blinded and were aware of the genotype of the animals prior to experimentation. No statistical method was applied to predetermine the same size of experimental groups. Appropriate animal health status, in accordance with our animal experimentation protocol, was used for exclusion criteria. No randomisation of the animals was used as experimental group assignment was primarily dependent on the appropriate animal genotype.

### Histological analysis

On the 7th day after injury, skin samples were obtained and fixed in 4% paraformaldehyde overnight, and then embedded in paraffin. Paraffin blocks were processed into 5-µm thick sections. Hematoxylin and eosin (H&E) staining was performed according to standard protocols, and morphometric analysis was conducted as previously described^51^. For immunofluorescence, protocols that are described elsewhere were applied^52^. Briefly, sections were deparaffined and incubated with primary antibody overnight at 4 °C after blocking with 5% BSA, and then visualized using secondary antibodies in blocking buffer for 1 h at room temperature. The primary monoclonal antibodies included anti-CD4, CD8, CD45, CD68, CD31, intercellular adhesion molecule-1 (ICAM-1) and vascular adhesion molecule-1 (VCAM-1) (Thermo Scientific, USA), AlexaFluor-488 -conjugated IgGs (Invitrogen, USA) secondary antibodies were used. DAPI (Beyotime, China) is used as a nuclear counterstain at a working concentration of 1 µg/mL. Immunofluorescence-stained and H&E-stained sections were imaged using a DMI 6000B microscope (Leica, Germany). Image analysis and quantifications were performed using ImageJ (National Institutes of Health, USA).

### Morphometric analysis

Morphometric analysis was performed on 5 µm sections obtained from the middle of the D7 wounds and were stained with H&E. Measurements of wound closure, HPE area (area of the epidermis measured from the point outside of the wound, at which it starts to become thicker than normal epithelium), and the length of newly formed wound epithelium (re-epithelialization) were carried out using ImageJ as previously described^20^.

### Measurement of leukocyte numbers and neovascularization at wound sites

The numbers of infiltrating CD4^+^, CD8^+^, CD45^+^ and CD68^+^ cells, and CD31^+^ ECs within the wound beds were enumerated in five randomly chosen visual fields of the sections, and the average of the selected five fields was calculated.

### ELISA measurement of serum TNF-α, IL-6, and IL-10 levels

The serum from the wounded mice was obtained, and TNF-α, IL-6, and IL-10 levels were measured by commercially available ELISA kits (Beyotime, China) according to the manufacturer’s recommendation.

### Cell culture and adenoviral infection

Human umbilical vein endothelial cells (HUVECs) were purchased from Lonza (Verviers, Belgium) and cultured in ECM (ScienCell Research Laboratories, USA) containing 10% fetal bovine serum (FBS) at 5% CO_2_, and 37 °C. Cells were used for experiments up to passage number 5. HUVECs were infected with adenoviral vectors containing rat calpastatin ([Ad-CAST] University of Buffalo, Buffalo, USA) or β-galactosidase ([Ad-gal] Vector Biolabs) at a multiplicity of infection of 100 plaque-forming units/cell. All experiments were performed after 24 h of adenoviral infection.

### Western blot analysis

HUVECs were collected and lysed with RIPA lysis buffer after treatments. The protein concentration was detected using a BCA kit (Beyotime, China). The protein levels of spectrin, ICAM-1, VCAM-1, β-catenin, IκB, and GAPDH (1:1000 dilutions, Cell Signaling Technology, USA) were determined by western blotting. Blots were scanned and quantified using an Odyssey imaging system (LI-COR Biosciences, USA). Quantified band intensities were normalized using GAPDH.

### Calpain activity assay

Calpain activity was assayed using a fluorometric kit (Abcam, Cambridge, UK) according to the manufacturer’s protocol. Calpain activity was analyzed using a fluorometer equipped with a 400-nm excitation filter and 505-nm emission filter.

### Cell viability and proliferation assays

Cell viability and proliferation were determined by using a CCK-8 kit (Kumamoto, Japan) and an EdU staining kit (RiboBio, China), respectively. Briefly, HUVECs were infected with Ad-CAST or Ad-gal, and then seeded onto culture plates. After treatment with 10 ng/mL TNF-α(Lonza, Verviers, Belgium) for 6 h, the absorbance was detected at 450 nm according to manufacturer instructions for the CCK-8 kit. For the EdU assay, the proliferation of HUVECs was detected with the Cell-Light EdU Apollo 488 In Vitro Imaging Kit (RiboBio, China) according to the manufacturer’s protocol. Briefly, HUVECs were seeded into 12-well plates at the density of 5 × 10^4^/well. After various treatments, the cells were stained with 50 μmol/L EdU for 2 h, fixed with 4% paraformaldehyde, and incubated with 2 mg/mL glycine for 5 min and 0.5% Triton X-100 in PBS for 10 min. Then, the cells were stained with 5 μg/mL DAPI and manually counted in five random high power fields (×100) in each well.

### Transwell assay

Migration analyses were carried out using the transwell chambers, which included upper and lower chambers separated by a polycarbonate filter (8 μm pore size). HUVECs were collected and resuspended in ECM without fetal bovine serum (FBS). Cells were seeded at 1 × 10^4^ cells/100 μL in the upper compartments of a 24-well plate, and 500 μL of medium containing 10% FBS was added into the lower chamber. The chambers were incubated at 37 °C, in 5% CO_2_ for 12 h. Cells in the lower chamber were stained with DAPI, and manually counted in five random high power fields (×200) in each well.

### Wound-healing assay

HUVECs were seeded in 12-well plates and cultured until at least 90% confluence. Wounds were made with a sterile pipette tip. After the dislodged cells were washed away, the cells were recultured with FBS-free media. Wounds were photographed at the initial timepoint and 12 h postwounding at the same location. The extent of healing was defined as the difference between the original and remaining wound areas, and is expressed as a percentage of the original area.

### Tube formation assay

The angiogenic ability of HUVECs was assessed by a tube formation assay as described previously^18^. Tube formation was determined by measuring tube length and mesh number.

### Statistical analysis

All data were analyzed by using Prism 7.0 software. One-or two-way ANOVA followed by Newman–Keuls tests were performed for multigroup comparisons, as appropriate. Student’s *t*-test was used for comparisons between two groups. A value of *p* < 0.05 was considered statistically significant.

## Supplementary information

Supplementary Information

Supplementary Information 2

Supplementary Information 3

Supplementary Information 4

Supplementary Information 5
