# Supplementary material for: Targeted inhibition of endothelial calpain delays wound healing by reducing inflammation and angiogenesis
Source: Cell Death Dis. 2020 Jul 14;11(7):533. doi: 10.1038/s41419-020-02737-x (PMC7360547; doi:10.1038/s41419-020-02737-x)
Supplement: Supplementary file 1 — Supplementary Information [file 41419_2020_2737_MOESM1_ESM.docx]

| Cre-F | GCGGTCTGGCAGTAAAAACTATC |
| --- | --- |
| Cre-R | GTGAAACAGCATTGCTGTCACTT |

Table 1 primer sequnence of cre
