# Supplementary material for: Targeted inhibition of endothelial calpain delays wound healing by reducing inflammation and angiogenesis
Source: Cell Death Dis. 2020 Jul 14;11(7):533. doi: 10.1038/s41419-020-02737-x (PMC7360547; doi:10.1038/s41419-020-02737-x)
Supplement: Supplementary file 2 — Supplementary Information 2 [file 41419_2020_2737_MOESM2_ESM.docx]

Supplementary figure legends

**sFigure 1**. Mouse genotype was determined using PCR of genomic tail DNA. Lane 2, 3, 4, 6, 12, 13: the expression of cre in the KO mice.

**sFigure 2**. Effects of Calpain Inhibitor I (ALLN) on cell migration and healing. Cultured HUVECs were incubated with or without ALLN (10 μM) in the presence of TNF-α (10 ng/mL) or vehicle. Representative images showed migration (A) and wound healing (B) of endothelial cells (scale bar, 100 μm). Bar graph showed results of quantitative analysis for migrated cells and the percentage of healing (C and D). Results are expressed as mean ± SD from four independent cell batches. **P*<0.05, vs con + vehicle; #*P*<0.05, vs con + TNF-α.

**sFigure 3**. ALLN treatment inhibited TNF-ɑ-induced tube formation and cell viability of HUVECs. Cultured HUVECs incubated with or without ALLN (10μM) in the presence of TNF-α (10 ng/mL) or vehicle. (A) Representative images show tube formation (scale bar, 200 μm). (B) Quantitative data of endothelial tube formation, as determined by tube length. (C) Cell viability was detected by CCK-8 assay. Results are expressed as mean ± SD from four independent cell batches. **P*<0.05, vs con + vehicle; #*P*<0.05, vs con + TNF-α.
