# Supplementary figures and images for: Targeted inhibition of endothelial calpain delays wound healing by reducing inflammation and angiogenesis
Source: Cell Death Dis. 2020 Jul 14;11(7):533. doi: 10.1038/s41419-020-02737-x (PMC7360547; doi:10.1038/s41419-020-02737-x)

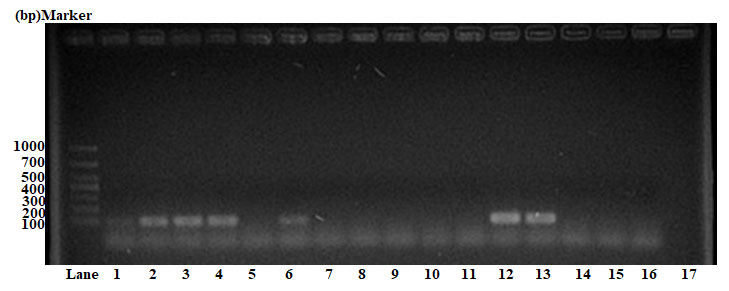

Supplement: Supplementary file 3 — Supplementary Information 3 [file 41419_2020_2737_MOESM3_ESM.png]

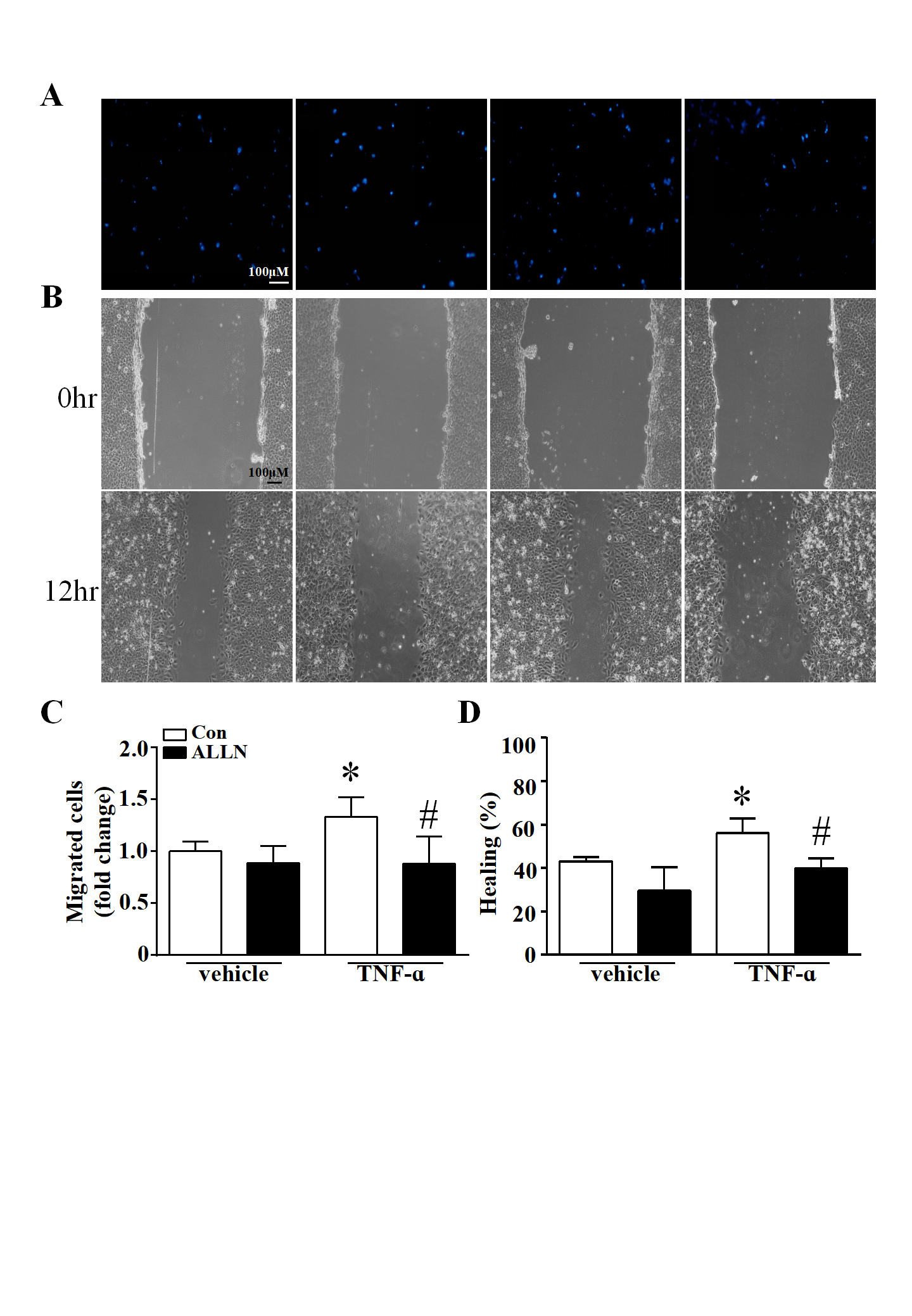

Supplement: Supplementary file 4 — Supplementary Information 4 [file 41419_2020_2737_MOESM4_ESM.png]

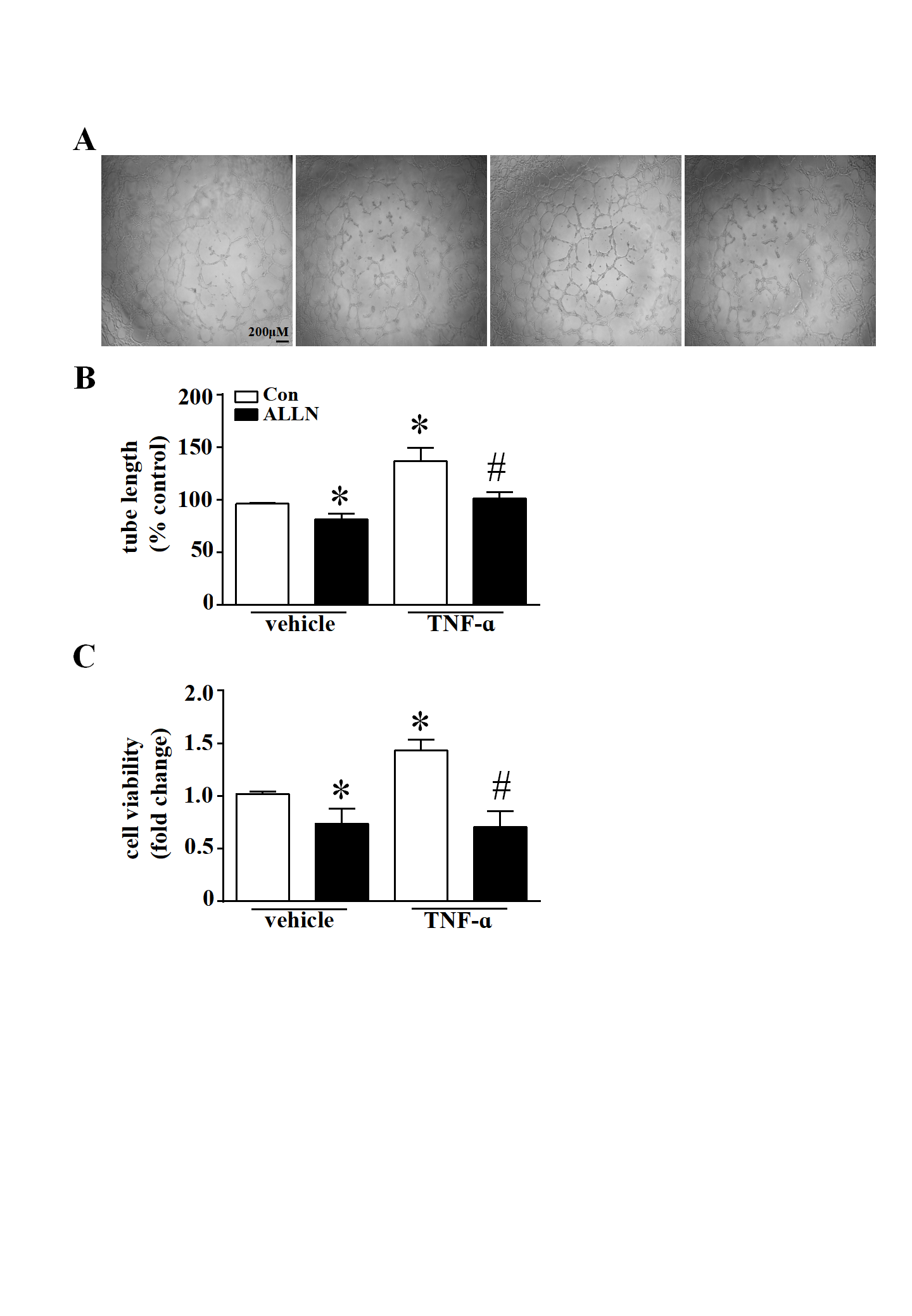

Supplement: Supplementary file 5 — Supplementary Information 5 [file 41419_2020_2737_MOESM5_ESM.png]
